# The Role of Preoperative Parenteral Nutrition

**DOI:** 10.3390/nu12051320

**Published:** 2020-05-06

**Authors:** Narisorn Lakananurak, Leah Gramlich

**Affiliations:** 1 Division of Clinical Nutrition, Department of Medicine, Faculty of Medicine, Chulalongkorn University, Bangkok 10330, Thailand; 2 Division of Gastroenterology, Department of Medicine, Faculty of Medicine and Dentistry, University of Alberta, Edmonton, AB T6G 2R3, Canada; lg3@ualberta.ca

**Keywords:** parenteral nutrition, surgery, preoperative period, nutrition support, nutrition assessment

## Abstract

Malnutrition is associated with poor surgical outcomes, and therefore optimizing nutritional status preoperatively is very important. The purpose of this paper is to review the literature related to preoperative parenteral nutrition (PN) and to provide current evidence based guidance. A systemic online search of PubMed, Medline, and Cochrane Databases from January 1990 to February 2020 was done. Sixteen studies were included in this narrative review, including four meta-analyses and twelve clinical trials. The majority of studies have demonstrated benefits of preoperative PN on postoperative outcomes, including reduced postoperative complications (8/10 studies) and postoperative length of stay (3/4 studies). Preoperative PN is indicated in malnourished surgical patients who cannot achieve adequate nutrient intake by oral or enteral nutrition. It can be seen that most studies showing benefits of preoperative PN often included patients with upper gastrointestinal cancer and inflammatory bowel disease (10/12 studies), which gastrointestinal problems are commonly seen and enteral nutrition may be not feasible. When preoperative PN is indicated, adequate energy and protein should be provided, and patients should receive at least seven days of PN prior to surgery. The goal of preoperative PN is not weight regain, but rather repletion of energy, protein, micronutrients, and glycogen stores. Complications associated with preoperative PN are rarely seen in previous studies. In order to prevent and mitigate the potential complications such as refeeding syndrome, optimal monitoring and early management of micronutrient deficiencies is required.

## 1. Introduction

Malnutrition in patients undergoing surgery is common. The incidence of malnutrition in surgical patients has been reported to range from 23–33% depending on type of surgery and nutrition assessment tool used [1,2]. Malnutrition is commonly seen in surgical patients with an underlying illness such as malignancy, chronic organ failure, and inflammatory bowel disease [3]. Moreover, the catabolic impact of surgery itself can negatively impact nutrition status. Surgery, like any injury, can result in release of stress hormones (e.g., cortisol, catecholamines, and glucagon) and inflammatory cytokines (e.g., tumor necrosis factor alpha, interleukins 1 and 6) [4]. Inflammation and this enhanced metabolic stress response may affect immune function, increase loss of muscle mass, and exacerbate malnutrition [5]. 

Numerous studies have demonstrated a clear association between preoperative malnutrition and poor surgical outcomes, including increased length of hospital stay, delayed wound healing, and increased infectious complications [6]. A study in 1244 patients undergoing surgery showed that malnutrition was significantly associated with increased length of hospital stay (13 days vs. 7 days) and increased postoperative complications with an odds ratio (OR) of 1.43 (95% confidence interval (CI) 1.16–1.76, *p* = 0.0006) [2]. 

Appropriate nutrition care prior to surgery is vital. It should include nutrition risk screening and formal assessment in those who screen positive for malnutrition [7]. In malnourished patients who are unable to meet nutrient requirements orally, including the use of oral nutrition supplements (ONS), parenteral nutrition (PN) should be considered. PN in these patients allows repletion of acute nutrient deficiencies and prevents further nutrition deficit development—it can narrow the nutritional gap in those who are facing a surgical procedure and in those recovering from surgery. PN provides an adequate and reliable amount of macronutrients and micronutrients, and the intravenous route of administration of nutrients may also allow for rapid improvement in nitrogen balance, increased muscle mass, faster recovery from surgery, improved immune function, and a decrease in the number of general and infectious complications [8]. Biologic plausibility has been demonstrated in recent studies. Preoperative PN for 12 h before surgery stimulated both protein transcription and translation, and reduced autophagy and lysosomal degradation of protein in patients undergoing major abdominal surgery [9,10]. In addition, PN may augment the immune system, including lymphocyte proliferation and activity in surgical patients [11,12].

Since the introduction of PN to the clinical practice, substantial clinical studies have evaluated the role of PN in patients undergoing surgery. The aim of this present study is to narratively review of the relevant literatures regarding preoperative PN over the past 30 years as well as to provide evidence-based and up-to-date guidance regarding the role of this nutritional treatment strategy. 

## 2. Materials and Methods

This qualitative, narrative review on the role of preoperative PN was conducted through a systemic online search of PubMed, Medline, and Cochrane Databases from January 1990 to February 2020. The following terms and keywords were used: (parenteral Nutrition or PN) AND preoperative AND surgery. Reference lists were hand searched for additional relevant studies.

Inclusion criteria for this study are as follows: (1) systematic reviews, meta-analyses, randomized controlled trials (RCTs), prospective studies, retrospective studies; (2) study populations limited to adult humans. Comments, conference abstracts, editorials, and studies published in languages other than English were excluded. Review articles retrieved during the literature search were hand searched to identify any further articles of relevance. In the case of continuing or duplicate studies, only the most recent publications were used.

**Figure 1:**
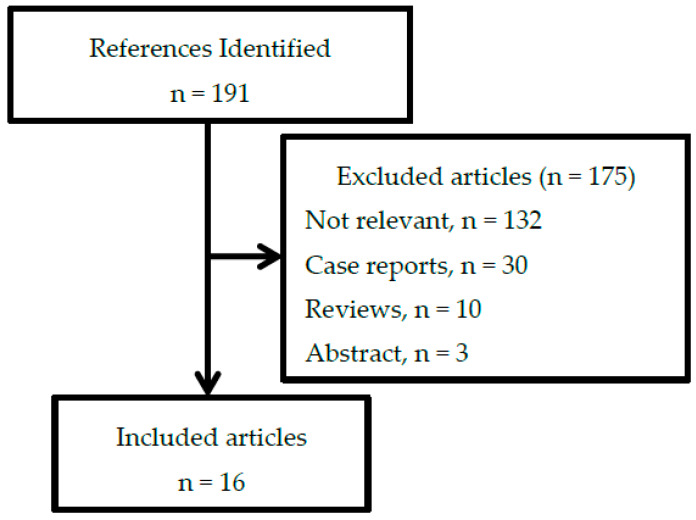
Flow diagram of the identified, excluded, and included articles in the narrative review.

A total of 191 articles were retrieved, of which 16 studies were included in this narrative review. The most common reason to exclude the articles is irrelevant, primarily because they did not discuss preoperative PN (Figure 1).

## 3. Results

There were four systematic reviews and meta-analyses evaluating the role of preoperative PN. Two reviews focused on Crohn’s disease patients [13,14] while the others assessed patients who underwent general surgery [15] and gastrointestinal surgery [16]. Furthermore, 12 studies (5 RCTs, 4 prospective studies, and 3 retrospective studies) were included in this narrative review [17,18,19,20,21,22,23,24,25,26,27,28]. Five of these studies were also included in previous systematic reviews and meta-analyses (2 studies in Heyland et al. [15], 1 study in Burden et al. [16], 3 studies in Grass et al. [13], and 1 study in Brennan et al. [14]). There is diversity among research participants, including nutrition status and types of surgery. Two-thirds of the studies (8/12) included only malnourished patients. Two studies enrolled patients with mixed nutrition status (29% malnutrition in the study by Von Meyenfeldt et al. and 22.2% malnutrition in the study by Grivceva et al.) [18,22]. Nutrition status was not mentioned in the rest trials (2 studies) [24,25]. Almost 60% of the studies (7/12) enrolled patients undergoing gastrointestinal surgery, followed by patients with inflammatory bowel disease (4/12, 33.3%). Thoracic surgery and various major surgical procedures were investigated in only one study [17,28]. Table 1 summarizes the details of 12 preoperative PN studies published between January 1990 and February 2020.

### 3.1. Benefits of Preoperative Parenteral Nutrition

Most studies evaluated benefits of preoperative PN with 3 main outcomes which were postoperative complications, mortality, and length of stay (LOS). 

#### 3.1.1. Postoperative Complications

Preoperative PN is associated with a significant reduction in postoperative complications in malnourished surgical patients, especially in those with severe malnutrition. Postoperative complications usually included infectious complications (intra-abdominal abscess, would infection, pneumonia, and sepsis) and non-infectious complications (anastomotic leak, wound dehiscence, organ failure, and thromboembolism). 

A meta-analysis in 2001 by Heyland et al., containing 27 RCTs (2,907 patients), showed that PN was associated with a reduction in complication rates in surgical patients (relative risk (RR) = 0.81, 95% CI, 0.65 to 1.01, *p* = 0.06) and a significant reduction was only demonstrated when studies in malnourished patients were analyzed (RR = 0.52, 95% CI, 0.30 to 0.91) [15]. There was a significant reduction in major complications associated with the use of preoperative PN in gastrointestinal surgery patients (RR 0.64; 95% CI: 0.46–0.87) in another meta-analysis in 2012 [16]. Moreover, in a recent meta-analysis of Crohn’s disease patients, preoperative PN showed a trend toward the reduction of complications with an OR of 0.65 (95% CI: 0.23–1.88, *p* = 0.43). However, this trend did not reach statistical significance [14].

PN prior to surgery was found to reduce postoperative complications in 8 out of 10 studies that evaluated this outcome. However, the benefit was only significantly demonstrated in a severe malnutrition subgroup in 2 out of these 8 studies. A large RCT from the 1990’s of preoperative PN in 395 surgical patients who required laparotomy or non-cardiac thoracotomy showed that there were significantly lower non-infectious complications in the PN group (5% vs. 43%, *p* = 0.03) in severely malnourished patients defined by subjective global assessment (SGA) C or Nutrition Risk Index (NRI) < 83.5 [17]. Additionally, lower septic complications were illustrated in patients with more than 10% weight loss in another RCT (5.6% vs. 81.8%, *p* < 0.05) [18]. In contrast, one study by Yao et al., comprising of severely malnourished Crohn’s disease patients, demonstrated a non-significant lower complication rate in the PN group compared to the control group (37.5% vs. 43.8%, *p* = 0.86) [20]. In addition, a study by Salinas et al. showed higher total complications in the PN group compared to the control group (50% vs. 35.2%, *p* = 0.047) [25]. It can be observed that the percentage of studies showing this benefit before the publication of the NICE-SUGAR study in 2009 [29] was less than the percentage after the publication (50% (3/6) vs. 75% (3/4)). This may be related to strategies to optimize glycemic control and to minimize risk of hyperglycemia in patients on PN.

#### 3.1.2. Mortality

The effect on mortality rate was not observed in the meta-analyses and the majority of studies regarding preoperative PN. Only one prospective study in malnourished gastric and colorectal cancer patients by Wu et al. in 2006 was able to demonstrate a lower mortality rate in the perioperative PN group (7 days preoperative PN and 7 days postoperative PN), compared to the control group (2.1% vs. 6%, *p* = 0.003) [21].

#### 3.1.3. Length of Hospital Stay

LOS was evaluated in 7 studies. The benefit of reduced total hospital LOS still remains controversial. Preoperative PN is related to a significantly longer total LOS in one study (median LOS = 33 days vs. 27 days, *p* < 0.001) [19], a shorter total LOS in one study (median LOS = 34 days vs. 52 days, *p* < 0.014) [21], and no significant difference in total LOS in the other 3 studies [18,22,27]. The longer total LOS may be due to the need for hospitalization in order to receive PN prior to surgery, whereas the shorter LOS could be explained by the reduction in postoperative complications. On the contrary, most studies (3/4 studies) demonstrated a significant reduction in postoperative LOS for approximately 4–14 days in patients receiving preoperative PN [23,27,28]. A cohort study by Jie et al. in 2012 illustrated that mean total LOS was not significantly different (26.2 days vs. 25.7 days, *p* = 0.806), while mean postoperative LOS was significantly shorter (13.7 days vs. 17.9 days, *p* = 0.018) in patients with preoperative PN [27]. 

#### 3.1.4. Other Outcomes

A study in 32 patients with Crohn’s disease by Yao et al. in 2005 illustrated that mean body mass index (BMI) significantly increased (13.9 kg/m^2^ to 15.3 kg/m^2^, *p* = 0.02) and mean serum immunoglobulin significantly decreased (133 ± 16 mg/dL to 105 ± 29 mg/dL, *p* = 0.02) in patients receiving 1 week preoperative PN and 3 weeks postoperative PN [20]. However, the increase of BMI could be attributable to fluid gains from PN rather than an increase in lean body mass. The author explained that PN can ameliorate the humoral immunity by repairing bowel integrity and promoting recovery of the disease by avoiding the stimulation of food. 

### 3.2. Preoperative Nutrition Assessment

The benefits of perioperative nutritional therapy, including preoperative PN, are usually observed in malnourished surgical patients, and therefore nutrition status should be assessed in all patients before major surgery [30].

Various nutrition screening and assessment methods were used in previous studies. The most common criteria or tools for diagnosis of malnutrition are low serum albumin (3 studies), low BMI (3 studies), and weight loss > 10% (3 studies), followed by SGA in 2 studies. Each of other methods, including Nutrition Risk Index (NRI), Nutrition Index (NI), nutrition risk screening (NRS), prealbumin, total lymphocyte count, mid-arm circumference, and triceps skinfold thickness, is used in one study. 

In 2017, the European Society for Clinical Nutrition and Metabolism (ESPEN) published clinical practice guidelines on clinical nutrition in surgery. The diagnosis of malnutrition is based on two options: (1) BMI < 18.5 kg/m^2^, (2) combined: weight loss >10% or >5% over 3 months and reduced BMI (<20 kg/m^2^ or <22 kg/m^2^ in patients aged younger and older than 70 years, respectively) or a low fat free mass index (FFMI, <15 kg/m^2^ in females and <17 kg/m^2^ in males). The guidelines recommend to define patients at severe nutrition risk by one of the following criteria: (1) weight loss >10–15% within 6 months, (2) BMI < 18.5 kg/m^2^, (3) SGA grade C or NRS > 5, and (4) preoperative serum albumin < 30 g/L (without evidence of liver or kidney dysfunction). Each recommended tool is a prognostic factor for postoperative complications and is associated with impaired nutrition status [30]. Serum albumin is not a good indicator of nutritional status since it does not change in response to changes in nutrient intake and is neither sensitive nor specific enough to evaluate malnutrition [31,32]. Given that albumin is a negative acute phase protein, it is rather a marker for severity of inflammation and is associated with morbidity and mortality of disease [31]. A systematic review of 15 studies in elderly general surgery patients showed that preoperative albumin concentration was a significant predictive parameter for postoperative outcomes, including postoperative complications, mortality, and LOS [33].

To improve the clinical care and outcomes, nutrition care pathway should be implemented in all surgical patients. It includes not only nutrition screening and assessment by the validated tools but also nutrition treatment and monitoring according to severity of malnutrition. This process allows identification of those who may benefit from nutrition treatment strategies. One example of nutrition care pathway is the Integrated Nutrition Pathway for Acute Care (INPAC), which is a validated pathway using in Canada to improve the prevention, detection, and treatment of malnutrition and thus influence clinical care and outcomes [34]. The Canadian Nutrition Screening Tool (CNST) is used for nutrition screening and patients identified to be at risk of malnutrition by the screening tool are classified of nutrition status using SGA. Comprehensive nutrition assessment by dietitians and specialized nutrition care including the use of PN are required for malnourished patients who are unable to achieve nutrient requirements by oral intake including the use of ONS [7].

### 3.3. Who May Benefit from Preoperative Parenteral Nutrition?

As previously mentioned, the benefits of preoperative PN were limited in patients without malnutrition. Hence, it should only be indicated for patients at risk for malnutrition who require surgery, especially in those with severe malnutrition.

Although early meta-analyses suggested the use of enteral nutrition (EN) was favorable compared to PN [35,36], recent studies in intensive care unit (ICU) identify that they are equivalent with respect to complications and outcomes if dosing is equivalent and indeed, patients on EN may get more complications. A 2018 meta-analysis of 16 RCTs in critical illness, including surgical patients, demonstrated that there were no clear clinical advantages of EN over PN in term of mortality, pneumonia, and length of hospital stay [37]. A large RCT in 2388 patients also showed that PN is neither more harmful than EN regarding mortality and infectious complications when protein and caloric intake were similar in both groups. However, patients in EN group experienced more hypoglycemia and vomiting [38]. Few studies have directly compared between preoperative EN and PN. In a study by Von Meyenfeldt et al., postoperative complications and mortality were not significantly different between patients receiving preoperative PN and EN. Interestingly, in those with >10% weight loss, there was a significant decrease in the number of patients developing intra-abdominal abscess in the PN group [18]. Optimizing intake by enteral feeding may be difficult and take more time than repletion by the intravenous route [39]. In addition, refusal of enteral feeding to avoid a nasal feeding tube is not uncommon [40,41]. Jie et al. examined the benefits of nutrition therapy prior to abdominal surgery. In this study, only 20.9% of patients achieved goal caloric intake with the use of EN whereas 79% of those with PN achieved goal nutrition support [27]. Some patients may experience feeding-associated gastrointestinal problems, such as vomiting, abdominal pain, abdominal distension, and diarrhea, especially in patients with preexisting gastrointestinal dysfunction [3]. It can be seen that studies showing benefits of preoperative PN often included patients with upper gastrointestinal cancer and inflammatory bowel disease (10/12 studies), which gastrointestinal problems are commonly seen. Therefore, the patients who are most likely to benefit from preoperative PN are actually those who are least likely able to tolerate EN because of these problems. 

### 3.4. Dose and Duration of Preoperative Parenteral Nutrition

The ESPEN guidelines recommend estimated energy and protein requirement with 25–30 kcal/kg/day and 1.5 g/kg/day, respectively [30,42]. All energy and protein intakes from oral, EN, and PN should be taken into account. The energy and protein targets from previous preoperative PN studies, which were associated with good postoperative outcomes, ranged from 20–35 kcal/kg/day and 1–1.6 g/kg/day, respectively. Adequate supply of micronutrients according to recommended dietary allowance (RDA) should be provided. Intravenous vitamins and trace elements are indicated in patients who are unable to achieve adequate requirement via enteral route. Early intervention to correct suspected or confirmed micronutrient deficiencies should be implemented in all patients prior to surgery. 

The duration of preoperative PN from previous reports ranged from 5 to 90 days. PN was prescribed within a range of 7–14 days preoperatively in a majority of studies (8/11). The recovery of physiological function and body protein can be achieved as early as 7 days after PN [43]. At present, there is no specific target for the duration of preoperative nutrition support, and the goal of preoperative nutrition is not to regain patients’ usual body weight but to acutely replete energy and protein storage, and micronutrient deficiencies, and to prevent further nutrition deficit in those at nutrition risk for the stress of surgery [44]. The longer duration of preoperative PN was found in a study demonstrating benefit of preoperative PN on postoperative complications in patients with Crohn’s disease (18–90 days) [24]. On the contrary, in patients with cancer, the duration was usually limited to 5–10 days prior to surgery. 

### 3.5. Complications and Monitoring of Preoperative Parenteral Nutrition

One of the most significant concerns about preoperative PN is its complications. However, preoperative PN is safe according to the previous studies. Complications of PN can be divided into catheter-related complications and PN-related complications. Catheter-related complications include complications of intravenous line insertion (e.g., pneumothorax, bleeding, and air embolism), catheter-related infections, line migration, and venous thrombosis. These complications are not common and relatively easy to detect. Appropriate catheter care is a measure to prevent infectious complications. There are 8 studies reporting catheter-related complications. Overall, catheter-related infection occurred in 0–10.7% of patients receiving preoperative PN, and there was no catheter-related infections in most studies (5/8 studies) [17,19,20,24,28]. The highest rate of infection was shown in a retrospective study in patients with ulcerative colitis, which preoperative PN was given in a relatively longer duration (7–28 days), compared to the other studies [25]. Other catheter-related complications were observed in 0–6.7% (pneumothorax), 0–1.8% (phlebitis), 0–1.6% (air embolus), and 0–0.5% (thrombosis) of patients. 

PN-related complications, including refeeding syndrome, electrolyte abnormalities, dysglycemia, volume overload, and liver injury, are also not commonly seen in patients with preoperative PN. Four studies evaluated these complications, and almost all of those studies illustrated no significant PN-related complications (3/4 studies). One patient (6.3%) experienced cholestatic liver injury in a study by Yao et al, which evaluated 7 days of preoperative PN in patients with Crohn’s disease [20].

In order to prevent and provide early treatment for complications, signs and symptoms of vascular access device complications and volume overload should be monitored daily during PN infusion. Blood for electrolytes, renal function, liver tests, and triglycerides should be checked daily until stable and then at least every week (more frequently if clinically significant metabolic abnormalities are found or patient is at risk for refeeding syndrome) [45]. The duration of at least 7 days before surgery allows enough time to address any electrolyte disturbance that may occur [6]. Glucose control before elective surgery is very important and beneficial in decreasing postoperative complications [46]. Therefore, blood sugar levels should be monitored and a target of less than 180 mg/dL is recommended [30]. 

### 3.6. Preoperative Parenteral Nutrition: New Directions

As preoperative PN may result in increased total LOS and hospital costs, home or outpatient PN is a strategy that needs to be explored in order to address the challenges associated with preoperative PN used to optimize surgical outcomes [3]. To the best of our knowledge, there is no study focusing on this strategy. The Outpatient Preoperative Parenteral Nutrition in malnourished surgical patients study (The OPPortuNity study, ClinicalTrials.gov Identifier: NCT03926949) is a pilot RCT, conducted in malnourished intra-abdominal and thoracic surgical patients (SGA B or C) to evaluate feasibility and outcomes (postoperative complications, postoperative LOS, quality of life, nutrition status, and readmission rate) of 5-to 10-day-preoperative PN in an outpatient setting. The patient recruitment is now underway. Patients in the study have been able to receive preoperative PN in an outpatient setting and this is able to narrow the gap of energy and protein intake from 46% to 85% of estimated energy requirement and from 39% to 100% of estimated protein requirement. They can complete PN administration without any complications. The use of total nutrient admixtures with high protein (32% by calories) and relatively limited in dextrose (26.2% by calories) in the study may be attributable to the feasibility and reduced risk of refeeding syndrome of outpatient preoperative PN. A comprehensive analysis of the final results will allow us to test this hypothesis and make conclusions.

There is evident that malnutrition and major surgery are related with reduced quality of life and impaired functional status [47,48]. Moreover, PN may positively impact on these outcomes especially in cancer patients [49,50]. However, the effects of preoperative PN on quality of life and patients’ functional status have not been evaluated in previous studies. Therefore, these outcomes should be assessed in further studies in order to provide a more comprehensive assessment of postoperative outcomes. 

In addition, body weight, serum albumin, or other nutrition parameters may not be good indicators for evaluation adequacy of preoperative PN since they can be affected by several factors (e.g., inflammation, fluid overload) and usually do not improve in a short period of time (7–14 days before surgery). Therefore, other specific targets to justify adequate preoperative PN are required. Finally, there are no studies that compare clinical outcomes between shorter (5–7 days) and longer (14 days or more) periods of preoperative PN. 

### 3.7. Limitations

Given that we excluded studies published in languages other than English, language bias may occur in this review. Despite several studies regarding preoperative PN after 1990, the studies usually vary in terms of population, nutrition status, types of surgery, dose, and duration of PN. Moreover, most studies contained a small sample size, which could indicate inadequate power to evaluate the outcomes. Thus, large randomized trials are needed for sufficient power to suggest stronger evidence. 

## 4. Conclusions

**Figure 2:**
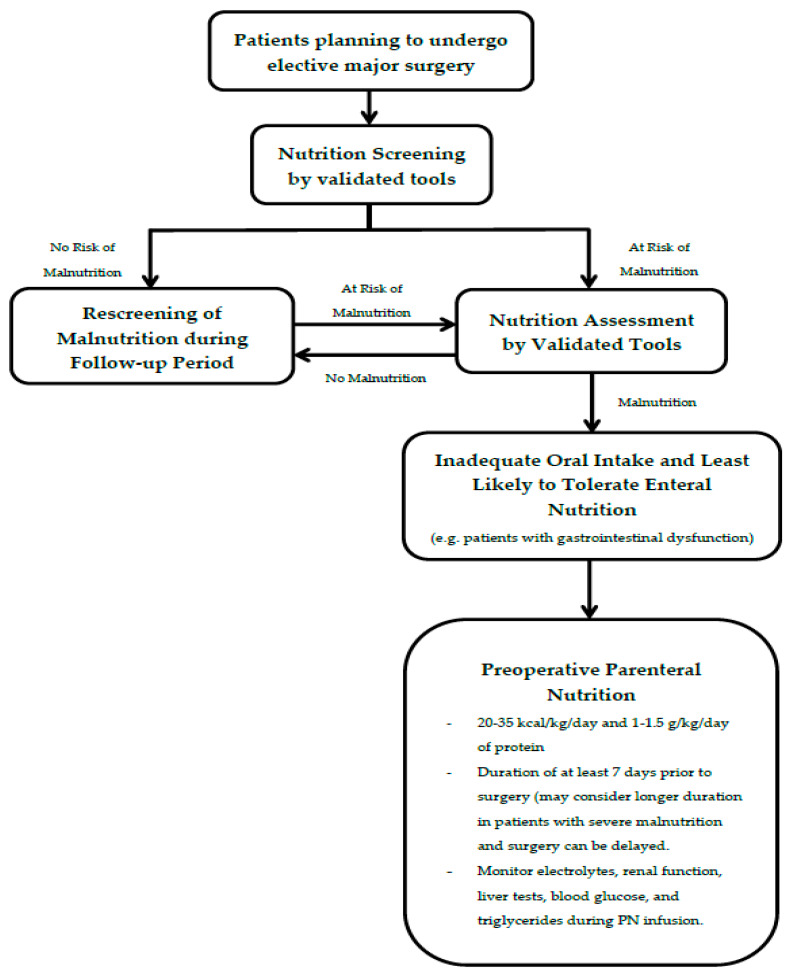
Summary of indication, dose, duration, and monitoring of preoperative parenteral nutrition.

Preoperative PN is associated with beneficial effect on several postoperative outcomes, including decreased postoperative complications and LOS. This treatment modality should be only indicated in malnourished surgical patients, and therefore nutrition screening and assessment by validated tools should be implemented in all patients who undergo elective surgery. The patients who are most likely to benefit from preoperative PN are those who are least likely able to tolerate oral intake and/or EN. This is particularly in patients with upper gastrointestinal cancer and inflammatory bowel disease. While there is no specific target for the duration of PN administration, PN duration of at least 7 days before surgery is recommended. Even though complications related to administration of preoperative PN are not common, appropriate monitoring and early management of those complications are necessary. Figure 2 summarizes indication, dose, duration, and monitoring of preoperative PN. 

## Figures and Tables

**Table 1:** Summarizes the details of 12 preoperative parenteral nutrition (PN) studies published between January 1990 and February 2020.

Study, Year	Study Design	Patients (Number)	Nutrition Status	Intervention		Postoperative Complications			Mortality			LOS (Days)			Remark
				Intervention	Control	Intervention	Control	*p*-Value	Intervention	Control	*p*-Value	Intervention	Control	*p*-Value	
1. Veterans Affairs Total Cooperative Study Group 1991	Randomized Control Trial	Thoracoabdominal surgery (395)	100% Malnutrition *	7–15 days preop and 3 days postop PN (with lipid)	No PN	49/192 (25.5%)	50/203 (24.6%)	>0.05	31/231 (13.4%)	24/228 (10.5%)	>0.05	N/A			Lower noninfectious complications in PN group (5% vs. 43%, *p* = 0.03) in patients with severe malnutrition (SGA C or NRI < 83.5)
2. Von Meyenfeldt et al. (1992)	Randomized Control Trial	Gastric cancer (29) Colorectal cancer (72)	29% Malnutrition (Nutrition index <1.31)	10 days preop PN (with lipid)	No PN	6/51 (11.8%)	7/50 (14%)	>0.05	2/51 (3.9%)	2/50 (4%)	>0.05	Mean total (SD) = 36.3 (17.7)	Mean total (SD) = 31.7 (22.1)	>0.05	Lower septic complication in PN group (5.6% vs. 81.8%, *p* < 0.05) in patients >10% weight loss
3. Bozzetti et al. 2000	Randomized Control Trial	Gastric cancer (74) Colorectal cancer (16)	100% Malnutrition (>10% weight loss in 6 months)	10 days preop and 9 days postop PN (with lipid)	No preop PN and 9 days postop PN (940 kcal + 85 g protein)	16/43 (37.2%)	27/47 (57.4%)	**0.03**	0/43 (0%)	5/47 (10.6%)	0.05	Median total (range) = 33 (18–161)	Median total (range) = 27 (15–103)	**<0.001**	
												Median postop (range) = 14 (7–143)	Median postop (range) = 14 (6–59)	0.98	
4. Yao et al. 2005	Prospective Study	Crohn’s disease (32)	100% Severe Malnutrition (BMI < 15 kg/m^2^)	1 week preop and 3 weeks postop PN (with lipid)	No PN	6/16 (37.5%)	7/16 (43.8%)	0.86	N/A			N/A			- BMI increased significantly in PN group (13.9 ± 0.6 to 15.3 ± 0.7 kg/m^2^ (*p* = 0.02)) - Serum IgM decreased significantly in PN group (133 ± 16 mg/dL to 105 ± 29 mg/dL, *p* = 0.02)
5. Wu et al. 2006	Prospective study	Gastric cancer (253) Colorectal cancer (215)	100% Malnutrition (SGA B or C)	7 days preop and 7 days postop PN (with lipid) (68%) or EN (32%)	Preop standard oral diet and postop hypocaloric PN	31/235 (13.2%)	64/233 (27.5%)	**0.012**	5/235 (2.1%)	14/233 (6%)	**0.003**	Median total = 34	Median total = 52	**0.014**	
6. Grivceva et al. 2008	Retrospective Study	Severe Crohn’s disease (63) and severe ulcerative colitis (27)	22.2% Malnutrition (BMI < 18.5 kg/m^2^)	Mean (SD) = 12.5 (5) days preop PN	No PN	N/A			N/A			Mean total (SD) = 18.9 (8.9)	Mean total (SD) = 18.9 (6.5)	0.98	
7. Wu et al. 2008	Retrospective study	Gastric cancer underwent TG (40) and SG (78)	100% Malnutrition (weight loss >10% in 6 months or albumin < 3 g/dL)	At least 5 days preop and postop PN until can eat normally (with lipid)	No PN	4/25 (16%) TG	10/15 (66.7%) TG	**0.002**	1/25 (4%) TG	4/15 (26.7%) TG	0.056	Mean postop (SD) = 21.3 (12.3)	Mean postop (SD) = 35.2 (25.1)	**0.024**	
						10/46 (21.7%) SG	14/32 (43.8%) SG	**0.048**	2/46 (4.4%) SG	4/32 (12.5%) SG	0.221	Mean postop (SD) = 14.5 (4.3)	Mean postop (SD) = 13.4 (2.9)	0.261	
8. Jacobson et al. 2012	Prospective Study	Moderate to severe Crohn’s disease (120)	N/A	18–90 days (mean 46 days) preop PN (with lipid)	No PN	0/15 (0%)	29/105 (27.6%)	**<0.05**	N/A			Mean postop (SD) = 17 (7)	N/A		
9. Salinas et al. 2012	Retrospective Study	Ulcerative colitis (235)	N/A	7–28 days (median 9 days) preop PN	No PN	28/56 (50%)	63/179 (35.2%)	**0.047**	1/56 (1.8%)	0/179 (0%)	0.238	N/A			
10. Kirkil et al. 2012	Randomized Control Trial	Gastric cancer (35) Colorectal cancer (40)	100% Malnutrition (SGA B or C)	7days preop PN (with lipid)	Immune-enhancing EN, Standard EN, and No EN/PN	N/A			N/A			N/A			The mean total antioxidant capacity significantly increased in immune-enhancing EN and EN groups.
11. Jie et al. 2012	Prospective study	Intra-abdominal surgery (512)	100% Malnutrition (NRS ≥ 5)	7 days preop and 7 days postop PN (with lipid) (73.4%) or EN (26.6%)	No EN and PN	11/43 (25.6%)	39/77 (50.6%)	**0.008**	0/43 (0%)	2/77 (2.6%)	0.536	Mean total = 26.2 (10.1)	Mean total LOS = 25.7 (12.7)	0.806	
												Mean postop = 13.7 (7.9)	Mean postop LOS = 17.9 (11.3)	**0.018**	
12. Ganaie et al. 2015	Randomized Control Trial	Various major surgical procedures (100)	100% Malnutrition **	Preop and postop PN	No PN	8/50 (16%)	15/50 (30%)	**<0.05**	3/50 (6%)	3/50 (6%)		Mean postop LOS = 20 (11)	Mean postop LOS = 26.52 (13.78)	**<0.05**	

* By Nutrition Risk Index ≤100 and/or any two of: 1. weight ≤ 95% of ideal body weight, 2. albumin ≤ 39.2 g/L, 3. prealbumin ≤ 186 mg/L. ** By weight loss > 10%, body mass index < 18.8 kg/m^2^ for males and <18.4 kg/m^2^ for females, triceps skinfold thickness < 10 mm in males and <13 mm in females, mid-arm circumference < 25 cm in males and <23 cm in females, serum proteins < 6.5 g/dl, albumin < 3.5 g/dl and TLC < 1500. Abbreviation: LOS, length of stay; PN, parenteral nutrition; SGA, subjective global assessment; NRI, nutrition risk index; SD, standard deviation; BMI, body mass index; TG, total gastrectomy; SG, subtotal gastrectomy; EN, enteral nutrition; NRS, nutrition risk score.

## References

[1] Ozkalkanli M.Y., Ozkalkanli D.T., Katircioglu K., Savaci S. (2009). Comparison of tools for nutrition assessment and screening for predicting the development of complications in orthopedic surgery. Nutr. Clin. Pract. Off. Publ. Am. Soc. Parenter. Enter. Nutr..

[2] Thomas M.N., Kufeldt J., Kisser U., Hornung H.M., Hoffmann J., Andraschko M., Werner J., Rittler P. (2016). Effects of malnutrition on complication rates, length of hospital stay, and revenue in elective surgical patients in the G-DRG-system. Nutrition.

[3] Abunnaja S., Cuviello A., Sanchez J.A. (2013). Enteral and parenteral nutrition in the perioperative period: State of the art. Nutrients.

[4] Traynor C., Hall G.M. (1981). Endocrine and metabolic changes during surgery: Anaesthetic implications. Br. J. Anaesth..

[5] Yeh D.D., Fuentes E., Quraishi S.A., Cropano C., Kaafarani H., Lee J., King D.R., DeMoya M., Fagenholz P., Butler K. (2016). Adequate Nutrition May Get You Home: Effect of Caloric/Protein Deficits on the Discharge Destination of Critically Ill Surgical Patients. JPEN J. Parenter. Enter. Nutr..

[6] Evans D.C., Martindale R.G., Kiraly L.N., Jones C.M. (2014). Nutrition optimization prior to surgery. Nutr. Clin. Pract. Off. Publ. Am. Soc. Parenter. Enter. Nutr..

[7] Keller H., Laur C., Atkins M., Bernier P., Butterworth D., Davidson B., Hotson B., Nasser R., Laporte M., Marcell C. (2018). Update on the Integrated Nutrition Pathway for Acute Care (INPAC): Post implementation tailoring and toolkit to support practice improvements. Nutr. J..

[8] Ward N. (2003). Nutrition support to patients undergoing gastrointestinal surgery. Nutr. J..

[9] Iresjo B.M., Engstrom C., Lundholm K. (2016). Preoperative overnight parenteral nutrition (TPN) improves skeletal muscle protein metabolism indicated by microarray algorithm analyses in a randomized trial. Physiol. Rep..

[10] Iresjo B.M., Engstrom C., Smedh U., Lundholm K. (2019). Overnight Steady-State Infusions of Parenteral Nutrition on Myosin Heavy Chain Transcripts in Rectus Abdominis Muscle Related to Amino Acid Transporters, Insulin-like Growth Factor 1, and Blood Amino Acids in Patients Aimed at Major Surgery. JPEN J. Parenter. Enter. Nutr..

[11] Celaya Perez S., Navarro M., Roman A., Salinas J.C., Larrad L., Lasierra M.P., Lozano Mantecon R. (1989). The effect of preoperative parenteral nutrition on the capacity of the immune response in malnourished patients (preoperative parenteral nutrition and immunity). Nutr. Hosp..

[12] Ooi S.E., Chen G.W., Chou C.T. (2004). Adequate nourishment through total parenteral nutrition treatment may augment immune function in patients with colon cancer. Arch. Med. Res..

[13] Grass F., Pache B., Martin D., Hahnloser D., Demartines N., Hubner M. (2017). Preoperative Nutritional Conditioning of Crohn’s Patients-Systematic Review of Current Evidence and Practice. Nutrients.

[14] Brennan G.T., Ha I., Hogan C., Nguyen E., Jamal M.M., Bechtold M.L., Nguyen D.L. (2018). Does preoperative enteral or parenteral nutrition reduce postoperative complications in Crohn’s disease patients: A meta-analysis. Eur. J. Gastroenterol. Hepatol..

[15] Heyland D.K., Montalvo M., MacDonald S., Keefe L., Su X.Y., Drover J.W. (2001). Total parenteral nutrition in the surgical patient: A meta-analysis. Can. J. Surg. J. Can. Chir..

[16] Burden S., Todd C., Hill J., Lal S. (2012). Pre-operative nutrition support in patients undergoing gastrointestinal surgery. Cochrane Database Syst. Rev..

[17] Veterans Affairs Total Parenteral Nutrition Cooperative Study Group (1991). Perioperative total parenteral nutrition in surgical patients. N. Engl. J. Med..

[18] Von Meyenfeldt M.F., Meijerink W.J., Rouflart M.M., Builmaassen M.T., Soeters P.B. (1992). Perioperative nutritional support: A randomised clinical trial. Clin. Nutr..

[19] Bozzetti F., Gavazzi C., Miceli R., Rossi N., Mariani L., Cozzaglio L., Bonfanti G., Piacenza S. (2000). Perioperative total parenteral nutrition in malnourished, gastrointestinal cancer patients: A randomized, clinical trial. JPEN J. Parenter. Enter. Nutr..

[20] Yao G.X., Wang X.R., Jiang Z.M., Zhang S.Y., Ni A.P. (2005). Role of perioperative parenteral nutrition in severely malnourished patients with Crohn’s disease. World J. Gastroenterol..

[21] Wu G.H., Liu Z.H., Wu Z.H., Wu Z.G. (2006). Perioperative artificial nutrition in malnourished gastrointestinal cancer patients. World J. Gastroenterol..

[22] Grivceva Stardelova K., Misevska P., Zdravkovska M., Trajkov D., Serafimoski V. (2008). Total parenteral nutrition in treatment of patients with inflammatory bowel disease. Prilozi.

[23] Wu M.H., Lin M.T., Chen W.J. (2008). Effect of perioperative parenteral nutritional support for gastric cancer patients undergoing gastrectomy. Hepato-Gastroenterol..

[24] Jacobson S. (2012). Early postoperative complications in patients with Crohn’s disease given and not given preoperative total parenteral nutrition. Scand. J. Gastroenterol..

[25] Salinas H., Dursun A., Konstantinidis I., Nguyen D., Shellito P., Hodin R., Bordeianou L. (2012). Does preoperative total parenteral nutrition in patients with ulcerative colitis produce better outcomes?. Int. J. Colorectal Dis..

[26] Kirkil C., Bulbuller N., Aygen E., Basbug M., Ayten R., Ilhan N., Ilhan Y.S., Akbulut S. (2012). The effect of preoperative nutritional supports on patients with gastrointestinal cancer: Prospective randomized study. Hepato-Gastroenterol..

[27] Jie B., Jiang Z.M., Nolan M.T., Zhu S.N., Yu K., Kondrup J. (2012). Impact of preoperative nutritional support on clinical outcome in abdominal surgical patients at nutritional risk. Nutrition.

[28] Ganaie A.R., Itoo M.S., Bhat G.M. (2015). Effects of perioperative parenteral nutrition on wound healing and hospital stay in surgical patients: A randomized controlled study. Int. J. Res. Med. Sci..

[29] Investigators N.-S.S., Finfer S., Chittock D.R., Su S.Y., Blair D., Foster D., Dhingra V., Bellomo R., Cook D., Dodek P. (2009). Intensive versus conventional glucose control in critically ill patients. N. Engl. J. Med..

[30] Weimann A., Braga M., Carli F., Higashiguchi T., Hubner M., Klek S., Laviano A., Ljungqvist O., Lobo D.N., Martindale R. (2017). ESPEN guideline: Clinical nutrition in surgery. Clin. Nutr..

[31] Marcason W. (2017). Should Albumin and Prealbumin Be Used as Indicators for Malnutrition?. J. Acad. Nutr. Diet..

[32] Loftus T.J., Brown M.P., Slish J.H., Rosenthal M.D. (2019). Serum Levels of Prealbumin and Albumin for Preoperative Risk Stratification. Nutr. Clin. Pract. Off. Publ. Am. Soc. Parenter. Enter. Nutr..

[33] Van Stijn M.F., Korkic-Halilovic I., Bakker M.S., van der Ploeg T., van Leeuwen P.A., Houdijk A.P. (2013). Preoperative nutrition status and postoperative outcome in elderly general surgery patients: A systematic review. JPEN J. Parenter. Enter. Nutr..

[34] Keller H.H., McCullough J., Davidson B., Vesnaver E., Laporte M., Gramlich L., Allard J., Bernier P., Duerksen D., Jeejeebhoy K. (2015). The Integrated Nutrition Pathway for Acute Care (INPAC): Building consensus with a modified Delphi. Nutr. J..

[35] Braunschweig C.L., Levy P., Sheean P.M., Wang X. (2001). Enteral compared with parenteral nutrition: A meta-analysis. Am. J. Clin. Nutr..

[36] Braga M., Gianotti L., Gentilini O., Parisi V., Salis C., Di Carlo V. (2001). Early postoperative enteral nutrition improves gut oxygenation and reduces costs compared with total parenteral nutrition. Crit. Care Med..

[37] Tian F., Heighes P.T., Allingstrup M.J., Doig G.S. (2018). Early Enteral Nutrition Provided Within 24 Hours of ICU Admission: A Meta-Analysis of Randomized Controlled Trials. Crit. Care Med..

[38] Harvey S.E., Parrott F., Harrison D.A., Bear D.E., Segaran E., Beale R., Bellingan G., Leonard R., Mythen M.G., Rowan K.M. (2014). Trial of the route of early nutritional support in critically ill adults. N. Engl. J. Med..

[39] Jankowski M., Las-Jankowska M., Sousak M., Zegarski W. (2018). Contemporary enteral and parenteral nutrition before surgery for gastrointestinal cancers: A literature review. World J. Surg. Oncol..

[40] Bozzetti F. (2011). Nutritional support in oncologic patients: Where we are and where we are going. Clin. Nutr..

[41] Bozzetti F., Braga M., Gianotti L., Gavazzi C., Mariani L. (2001). Postoperative enteral versus parenteral nutrition in malnourished patients with gastrointestinal cancer: A randomised multicentre trial. Lancet.

[42] Braga M., Ljungqvist O., Soeters P., Fearon K., Weimann A., Bozzetti F., ESPEN (2009). ESPEN Guidelines on Parenteral Nutrition: Surgery. Clin. Nutr..

[43] Hill G.L. (1994). Impact of nutritional support on the clinical outcome of the surgical patient. Clin. Nutr..

[44] Torgersen Z., Balters M. (2015). Perioperative nutrition. Surg. Clin. N. Am..

[45] Ayers P., Adams S., Boullata J., Gervasio J., Holcombe B., Kraft M.D., Marshall N., Neal A., Sacks G., Seres D.S. (2014). A.S.P.E.N. parenteral nutrition safety consensus recommendations. JPEN J. Parenter. Enter. Nutr..

[46] Dronge A.S., Perkal M.F., Kancir S., Concato J., Aslan M., Rosenthal R.A. (2006). Long-term glycemic control and postoperative infectious complications. Arch. Surg..

[47] Marin Caro M.M., Laviano A., Pichard C. (2007). Impact of nutrition on quality of life during cancer. Curr. Opin. Clin. Nutr. Metab. Care.

[48] Maillard J., Elia N., Haller C.S., Delhumeau C., Walder B. (2015). Preoperative and early postoperative quality of life after major surgery—A prospective observational study. Health Qual. Life Outcomes.

[49] Hasenberg T., Essenbreis M., Herold A., Post S., Shang E. (2010). Early supplementation of parenteral nutrition is capable of improving quality of life, chemotherapy-related toxicity and body composition in patients with advanced colorectal carcinoma undergoing palliative treatment: Results from a prospective, randomized clinical trial. Colorectal Dis. Off. J. Assoc. Coloproctol. Great Br. Irel..

[50] Jin Y., Yong C., Ren K., Li D., Yuan H. (2018). Effects of Post-Surgical Parenteral Nutrition on Patients with Gastric Cancer. Cell. Physiol. Biochem..

